# Reducing Dietary Sodium Intake among Young Adults in Ghana: A Call to Action

**DOI:** 10.3390/nu15163562

**Published:** 2023-08-12

**Authors:** Emmanuel Peprah, Prince Amegbor, Amos Laar, Bismark Akasoe, Yvonne Commodore-Mensah

**Affiliations:** 1 Department of Global and Environmental Health, NYU School of Global Public Health, 708 Broadway, New York, NY 10003, USA; pma311@nyu.edu; 2 Department of Population, Family and Reproductive Health, School of Public Health, University of Ghana, Legon, Accra P.O. Box LG13, Ghana; alaar@ug.edu.gh (A.L.); bismark.akasoe@gmail.com (B.A.); 3 Johns Hopkins School of Nursing, 525 N. Wolfe Street, Baltimore, MD 21205, USA; ycommod1@jhmi.edu

**Keywords:** evidence-based interventions, global health, implementation research, sodium intake, Africa, young adults, adolescents, cardiovascular disease, hypertension

## Abstract

The positive association between excessive dietary sodium intake, hypertension, and cardiovascular disease (CVD) has been widely investigated in observational studies and clinical trials. Reducing sodium intake is a proven strategy to prevent hypertension and the onset of CVD, a major cause of morbidity and mortality globally. Africa has the youngest population globally, which is key to the continent’s sustainable development. However, in Africa, the epidemics of hypertension and CVD negatively impact life expectancy and economic growth. Ghana, like other African countries, is no exception. The factors contributing to the increasing burden of CVD and excessive sodium consumption are multi-faceted and multi-level, including individual lifestyle, neighborhood and built environments, and socio-economic and health policies. Thus, the implementation of evidence-based interventions such as the World Health Organization Best Buys that target the multi-level determinants of sodium consumption is urgently needed in Ghana and other African countries. The aim of this commentary is to highlight factors that contribute to excessive sodium consumption. Second, the commentary will showcase lessons of successful implementation of sodium reduction interventions in other countries. Such lessons may help avert CVD in young adults in Ghana and Africa.

## 1. Introduction

Approximately 2.6 million deaths, representing 35% of all deaths in the Sub-Saharan Africa (SSA) region, are attributable to non-communicable diseases [1]. Cardiovascular disease (CVD) is a leading cause of non-communicable diseases burden and mortality, accounting for 22.9 million disability-adjusted life years [2,3]. Amidst the growing CVD epidemic, there has been a significant increase in elevated blood pressure—a major risk factor of CVD [2,4,5] among populations in SSA. Furthermore, the World Health Organization (WHO) African Region has one of the highest prevalence rates of hypertension globally, with an estimated 46% of the adult population (>25 years) affected [6]. The main factors contributing to the increasing epidemics of hypertension and CVD in the SSA region are dietary and nutritional transitions, including the consumption of high amounts of dietary sodium [7,8,9].

Studies show a significant linear relationship between dietary sodium intake and CVD risk [10]. For example, the risk of CVD increases up to 6% for every 1 g increase in dietary sodium intake [10]. Moreover, excessive sodium consumption (e.g., >5 g per day) is associated with earlier onset of hypertension, one of the strongest risk factors for CVD [11,12,13] and with adverse physiological outcomes (e.g., cardiac fibrosis and endothelial and microvascular dysfunctions) [14,15,16]. These adverse effects on cardiovascular functions contribute to changes in arterial blood pressure, leading to hypertension [14,15,16,17,18]. While sodium is necessary for normal cellular homeostasis, and in the regulation of fluid and electrolyte balance, excessive sodium intake is also associated with a higher risk of osteoporosis [19] and stomach cancer [20,21].

Current studies provide strong evidence supporting the correlation between sodium intake and blood pressure levels; they also indicate that excessive consumption of sodium is linked to various other negative health conditions, including metabolic, gastrointestinal, and bone disorders [22,23,24,25,26]. Research suggests that a high intake of sodium can disrupt metabolism through mechanisms such as leptin resistance, overproduction of fructose and ghrelin, and insulin resistance [23]. In a cohort study conducted by Takase et al. [24,25], it was observed that increased salt intake correlated with a higher number of metabolic disorders and an elevated risk of developing metabolic syndrome. Excessive salt consumption has been associated with reduced bone density, leading to chronic musculoskeletal disorders like osteoporosis [25]. Other studies have also established a connection between high sodium or salt intake and gastrointestinal disorders such as Inflammatory Bowel Disease [26]. They propose that excessive salt intake disrupts immune homeostasis, thereby contributing to the development of these gastrointestinal disorders [27].

## 2. Sodium Intake Is High amongst Ghanaians and African Populations

Approximately one-third of Ghana’s population has hypertension, with higher prevalence rates observed among men and urban populations [28,29,30]. Further, the growing prevalence of hypertension and CVD in Ghana is consistent with patterns observed in many African countries (including South Africa, Egypt, and Tanzania) [31,32]. Dietary sodium intake is high among Ghanaians [33], with women having a median value of 8.6 g/day (g/day) compared to 7.5 g/day among men. Higher sodium intake is observed among younger adults (persons aged below 50 years—9.7 g/day) compared to older adults (persons aged 50 years and above—8.1 g/day) [33]. The sodium intake levels among Ghanaians exceeds the WHO recommended level of 2 g (equivalent to 5 g of salt) per person per day for the prevention of CVDs [34]. 

Ghana has the lowest score of 1 on the Sodium Country Score card because it is the only country that has a national policy commitment to sodium reduction but lacks voluntary or mandatory policies, declaration of sodium on pre-packaged food, and all WHO sodium-related “Best Buys” [35]. Additionally, data on sodium intake among children and adolescents in Ghana is limited. Research in high-income countries indicates higher sodium intake among children and adolescents, contributes to the early onset of CVD morbidity and mortality [36,37,38]. Although raised blood pressure and CVD are typically present in adults, the origins begin in childhood. Blood pressure has been shown to follow a tracking pattern; children and adolescents who have blood pressure at the higher end of the blood pressure distribution are more likely to develop high blood pressure in adulthood [39,40]. The negative consequences of high blood pressure are well documented in adults, hence the need for strategies to reduce sodium intake in Ghanaians and especially target interventions to children and adolescents.

## 3. Relationships between Sodium Intake, Socio-Cultural and Environmental Factors

The “built environment”, defined as the human-made surroundings that influence overall community health and individual behaviors [41], shape sodium intake and risk factors for CVD. Because of the importance of the environment for moderating behavior, the built environment is recognized as one of the key components of social determinants of health. Current data indicates that the vital role of modifiable environmental and socioeconomic factors, including urban environmental characteristics (e.g., population density, urban form, neighborhood safety, and food environment), have an indirect effect on blood pressure [1,3]. While it is generally acknowledged that the nutrition transition from traditional African diets to western ultra-processed and fast foods is a major contributor of the growing hypertension epidemic, some traditional African cuisines and cooking practices also encourage high sodium intake [42,43]. It is estimated that a sizeable number of Ghanaians often add salt to prepared food at the table, which results in increasing their sodium intake [33]. Indeed, some traditional delicacies such as salted fish (koobi), salted cured fish (momoni), and salt-preserved meats (beef or pork feet) also contribute to high sodium-intake among Ghanaians [42,44]. These food processing practices [15,16] coupled with consumption of ultra-processed foods underlie the increasing cases of hypertension and CVD among Ghanaians, particularly urban populations in Accra [17,18]. Traditional cooking practices exist with parallel ecosystems containing other environmental influences that result in increased sodium intake. Nevertheless, the built environment and food landscape encountered by Ghanaians are similar to the daily circumstances of various populations in SSA.

### 3.1. Built Environment

Currently, evidence on the effects of the built environment on diet risk factors, particularly sodium intake, is limited. Studies on sodium intake and CVD, including hypertension, often frame the role of the built environment in the context of food environments. These studies have mainly concentrated on the distribution of fast-food chains and healthy food options (grocery shops) [45,46]. Nonetheless, the food environment in SSA context is complex with the co-occurrence of Western fast-food restaurants, locally owned restaurants (often referred to as chop bars), and street food vendors. The phenomenon has significantly increased the consumption of street foods, with 50% of consumers prefer consuming breakfast at the aforementioned urban locations in SSA [47]. This change in behavior of food consumption results in street food significantly contributing to the daily intake of sodium (38.4%) and fat (49.1%) among Ugandans with little content of healthy nutrients [47]. These trends also exist in other African countries, including Ghana. Although there is limited data on current national sodium consumption in Ghana, studies show that the processed foods are largest component of food items sold nationally [48,49]. The distribution and concentration of these different food environments varies across SSA urban landscape. Yet, there is limited knowledge and understanding of the spatial distribution of these diverse sources of sodium and their potential contribution to the heterogeneity in sodium intake among adolescents in Accra. Indirectly, healthy built environments that encourage active transportation and increased physical activities among urban residents also help reduce sodium levels through sweating [50,51]. Likewise, other built environment features, such as land-use pattern, population density, street connectivity, and blue and green spaces, may regulate sodium levels and reduce the risk of hypertension through encouraging healthy lifestyles or behaviors [50]. 

### 3.2. Food Environments

Described as the physical or figurative interface that mediates one’s food acquisition and consumption within the wider food system, food environments can present both risks and benefits to the nutritional and health outcomes of people [52]. While healthy food environments provide equitable access to healthy foods, unhealthy food environments contribute to promoting unhealthy diets and related sequelae. Many countries in Africa are contending with unhealthy food environments characterized by widespread availability and consumption of energy-dense, nutrient-poor, ultra-processed foods and beverages [53,54,55]. Urban environments, in particular, have significant influences on health behaviors, including dietary habits and lifestyle [9,10]. The advertisement of energy-dense, nutrient-poor foods that are high in salt, sugar, and harmful fat is a common feature in obesogenic food environments. Studies in Ghana show advertising of sugary, fatty, and salty snacks to be prevalent in settings with children [3,56,57]. 

## 4. Multi-Level Evidence-Based Interventions (EBIs) Are Needed to Reduce Sodium Intake

EBIs to prevent non-communicable diseases that target multiple risk factors and are delivered at multiple levels (i.e., individual/patient, family, community, system, and policy levels) are more effective compared to strategies that focus on only one level [58,59,60]. Examples of such interventions include the WHO Best Buys [58], which are interventions considered the most cost-effective and feasible for implementation. WHO Best Buys include: (1) reducing salt intake by implementing front-of-pack labelling, (2) promoting product reformulation, (3) providing lower sodium options in public institutions, and (4) behavioral change communication and mass media campaigns to reduce the intake of sodium. The WHO has set a global target goal of a 30% reduction in salt intake by 2025 to meet the recommended limit of less than 5 g per day for adults [61]. This goal is projected to save millions of lives in Africa.

## 5. EBIs That Can Mitigate Sodium Intake and Impact the Downstream Effects of Hypertension and CVD among Adults

The factors that contribute to the rapid increase in hypertension and CVD are multi-dimensional and multi-factorial, so novel solutions are needed. Existing strategies tend to focus on one dimension and hence often lack a comprehensive overview of how the different dimensions interact and contribute to the growth in high blood pressure and hypertension among urban populations in Africa. A holistic approach that captures the complexities of the interactions between the environment, socioeconomic, and individual factors are needed to fully understand the antecedents to the growing health inequalities among urban populations, particularly those in the WHO African region using Ghana as a model. For example, an EBI including social and behavior change communication was used by the United Nations Children’s Fund (UNICEF) to improve adolescent nutrition in various countries, including Indonesia [62], Ethiopia [63], and Ghana [64]. A recent systematic review of 41,448 participants indicates that multi-component social and behavior change communication interventions, including both health education and awareness campaigns, were effective in reducing sodium intake [65]. Notwithstanding, the implementation of social and behavior change communication solely will not reduce dietary sodium intake to the recommended levels [65] in African countries because of the relationships of sodium intake with various aforementioned environmental factors. It is essential to incorporate families, communities, community organizations, industry leaders, and government stakeholders in policy development [66] when developing multi-level intervention strategies to reduce sodium intake among populations. 

## 6. Lessons Learned from Implementing Evidence-Based Sodium Reduction Policies and Programs in Other Countries

The increase in cases of hypertension linked to sodium intake is a public health concern globally. Policies, regulations, and programs are needed to control salt consumption. In 2013, the WHO considered the contribution of sodium to the global burden of non-communicable diseases and set the 30% voluntary global target for reducing sodium intake [10]. Subsequently, the WHO Best Buys, which include a set of cost-effective strategies to reduce population salt intake, were recommended for implementation [29,30]. Currently, salt reduction policies and programs developed and implemented by governments, health organizations, and other stakeholders have been proven effective at reducing salt intake [30]. Implementation of salt reduction program in United Kingdom (UK) resulted in a reduction in the population mean salt intake by 15%, and in Nigeria, such interventions resulted in reduction in 24 h urinary sodium, systolic blood pressure, and diastolic blood pressure [67]. 

In the UK, cross-sector collaboration led to salt reduction beyond the 10% target set for food industries [32,68]. The UK example highlights that implementation of salt reduction policies and programs requires the collaboration of various stakeholders [69] to change the food environment. Other examples of this type of successful cross-sector collaboration include Hungary’s introduction of a tax in 2011 on packaged foods with high salt content, which resulted in a 26% reduction in salt intake over three years. Similarly, Finland’s 2011 tax on salty snacks resulted in a 20% reduction in sales of salty snacks. Finally, France’s 2013 tax on salty food products resulted a reduction in the salt content of the affected products [34,35,36].

In addition to taxation, voluntary approaches such as food labeling and education campaigns have been effective in raising awareness and changing behavior. However, mandatory approaches such as setting maximum levels of salt in food products have been more effective in achieving significant reductions in salt intake, as in the case of South Africa [37,68,69]. Other studies suggest gradual reduction in salt levels in food products as a more acceptable approach to consumers and the food industry [70,71]. This approach allows for gradual adaptation of taste preferences and product reformulation. 

Studies have also recommended monitoring and evaluation of salt reduction interventions as essential to assess effectiveness and identify areas for improvement for EBI. Regular monitoring motivates stakeholders to maintain the momentum of the EBI and ensure that targets are being met [38,39]. Furthermore, public engagement has been identified as essential for the success of salt reduction interventions. Finally, salt reduction policies and programs need to be sustainable to achieve long-term benefits. Regular review and adaptation of key EBIs are necessary to ensure that they remain relevant and effective.

## 7. Conclusions

Protecting young adults in Ghana and other African countries from premature CVD and various complications requires urgent action to lower sodium intake. Implementing EBIs for education campaigns to lower sodium intake can contribute to the prevention of CVD. Utilizing multi-level strategies that include assessing community/neighborhood-level built and food environments (e.g., assessment of sources of sodium including chop bars and restaurants that influence diet) is essential to decrease hypertension and the burden of CVD in African populations. Predicated on lessons learned from implementation of national sodium reduction programs and policies in Europe (i.e., UK, Hungary, Finland, and France) and South Africa, effective government policy to reduce sodium levels in food can reduce sodium intake at the population level. Indeed, a holistic government approach combined with evidence-based health promotion efforts could delay or prevent progression to overt non-communicable diseases (i.e., hypertension) over the course of the lifetime for adolescents and young adults where CVD disease burden is low, thus preventing premature death and disability once they reach adulthood. Finally, implementation of EBI to mitigate behavior change in Ghana [18] for adolescents and young adults could have a higher likelihood of success in improving health outcomes compared to adults because young adults are more amenable to behavior change strategies compared to adults. A greater understanding of the drivers of the dietary habits and environmental influences of dietary sodium intake among adolescents and young adults is needed before a pragmatic context-specific EBI to mitigate dietary sodium intake can be implemented.
